# Rerouting phytosterol degradation pathway for directed androst-1,4-diene-3,17-dione microbial bioconversion

**DOI:** 10.1007/s00253-023-12847-z

**Published:** 2024-02-01

**Authors:** Xia Ke, Jia-Hao Cui, Qi-Jie Ren, Tong Zheng, Xin-Xin Wang, Zhi-Qiang Liu, Yu-Guo Zheng

**Affiliations:** 1 National and Local Joint Engineering Research Center for Biomanufacturing of Chiral Chemicals, Zhejiang University of Technology, Hangzhou, 310014 People’s Republic of China; 2 Key Laboratory of Bioorganic Synthesis of Zhejiang Province, College of Biotechnology and Bioengineering, Zhejiang University of Technology, Hangzhou, 310014 People’s Republic of China

**Keywords:** Steroidal intermediate, Microbial biotransformation, Whole-cell biocatalyst, Promoter engineering, Catabolic switch, Byproduct elimination

## Abstract

**Abstract:**

Steroid-based drugs are now mainly produced by the microbial transformation of phytosterol, and a two-step bioprocess is adopted to reach high space–time yields, but byproducts are frequently observed during the bioprocessing. In this study, the catabolic switch between the C19- and C22-steroidal subpathways was investigated in resting cells of *Mycobacterium neoaurum* NRRL B-3805, and a dose-dependent transcriptional response toward the induction of phytosterol with increased concentrations was found in the putative node enzymes including ChoM2, KstD1, OpccR, Sal, and Hsd4A. Aldolase Sal presented a dominant role in the C22 steroidal side-chain cleavage, and the byproduct was eliminated after sequential deletion of *opccR* and *sal*. Meanwhile, the molar yield of androst-1,4-diene-3,17-dione (ADD) was increased from 59.4 to 71.3%. With the regard of insufficient activity of rate-limiting enzymes may also cause byproduct accumulation, a chromosomal integration platform for target gene overexpression was established supported by a strong promoter L2 combined with site-specific recombination in the engineered cell. Rate-limiting steps of ADD bioconversion were further characterized and overcome. Overexpression of the *kstD1* gene further strengthened the bioconversion from AD to ADD. After subsequential optimization of the bioconversion system, the directed biotransformation route was developed and allowed up to 82.0% molar yield with a space–time yield of 4.22 g·L^−1^·day^−1^. The catabolic diversion elements and the genetic overexpression tools as confirmed and developed in present study offer new ideas of *M. neoaurum* cell factory development for directed biotransformation for C19- and C22-steroidal drug intermediates from phytosterol.

**Key points:**

*• Resting cells exhibited a catabolic switch between the C19- and C22-steroidal subpathways.*

*• The C22-steroidal byproduct was eliminated after sequential deletion of opccR and sal.*

*• Rate-limiting steps were overcome by promoter engineering and chromosomal integration.*

**Supplementary information:**

The online version contains supplementary material available at 10.1007/s00253-023-12847-z.

## Introduction

The large family of steroids includes numerous active pharmaceutical ingredients that are widely used in medicine, with immunomodulatory, diuretic, anti-inflammatory, antitumor, and contraceptive effects (Donova and Egorova 2012; Gupta and Mahajan 2019). Instead of the traditional chemical route, which often involves toxic heavy metal catalysts, microbial biotransformation of phytosterol has become a dominant route for the synthesis of steroidal intermediates in recent years. Some strains of the genus *Mycolicibacterium* (basonym: *Mycobacterium*) can utilize phytosterol as the sole carbon source by truncating the side chain and modifying the gonane nucleus (Donova et al. 2005; Sukhodolskaya et al. 2007; Zhao et al. 2021). The key enzymes involved in the phytosterol degradation pathway were gradually characterized (Fernandez-Cabezon et al. 2017; Kreit 2019). Combined with advanced target gene deletion methods (Yao et al. 2013), steroidal intermediates with specific structural modification could be simply obtained by one-step biotransformation using bioengineered *M. neoaurum* strains (Su et al. 2018; Xiong et al. 2020).

The well-recognized industrial strain *M. neoaurum* NRRL B-3805, which is characterized by its outstanding capability of sterol C19 side-chain cleavage, has been widely applied in the industrial biosynthesis of 4-androstene-3,17-dione (AD) and 1,4-androstediene-3,17-dione (ADD) (Mancilla et al. 2018). A two-step biotransformation process is usually adopted using resting cells as biocatalysts, with advantages including non-sterile conditions, convenient product separation, and high volumetric productivities (Chang et al. 2020; Liu et al. 2018). With the aim of high productivity and simple downstream processing, previous efforts were mainly devoted to the optimization of fermentation/biotransformation conditions, including parameters such as dissolved oxygen (Zhang et al. 2021), rotation speed, pH, temperature, and electron transfer (Chang et al. 2020; Zhou et al. 2019a, 2020). However, byproducts were still observed in addition to the designed bioconversion route from phytosterol to the end product.

**Fig. 1:**
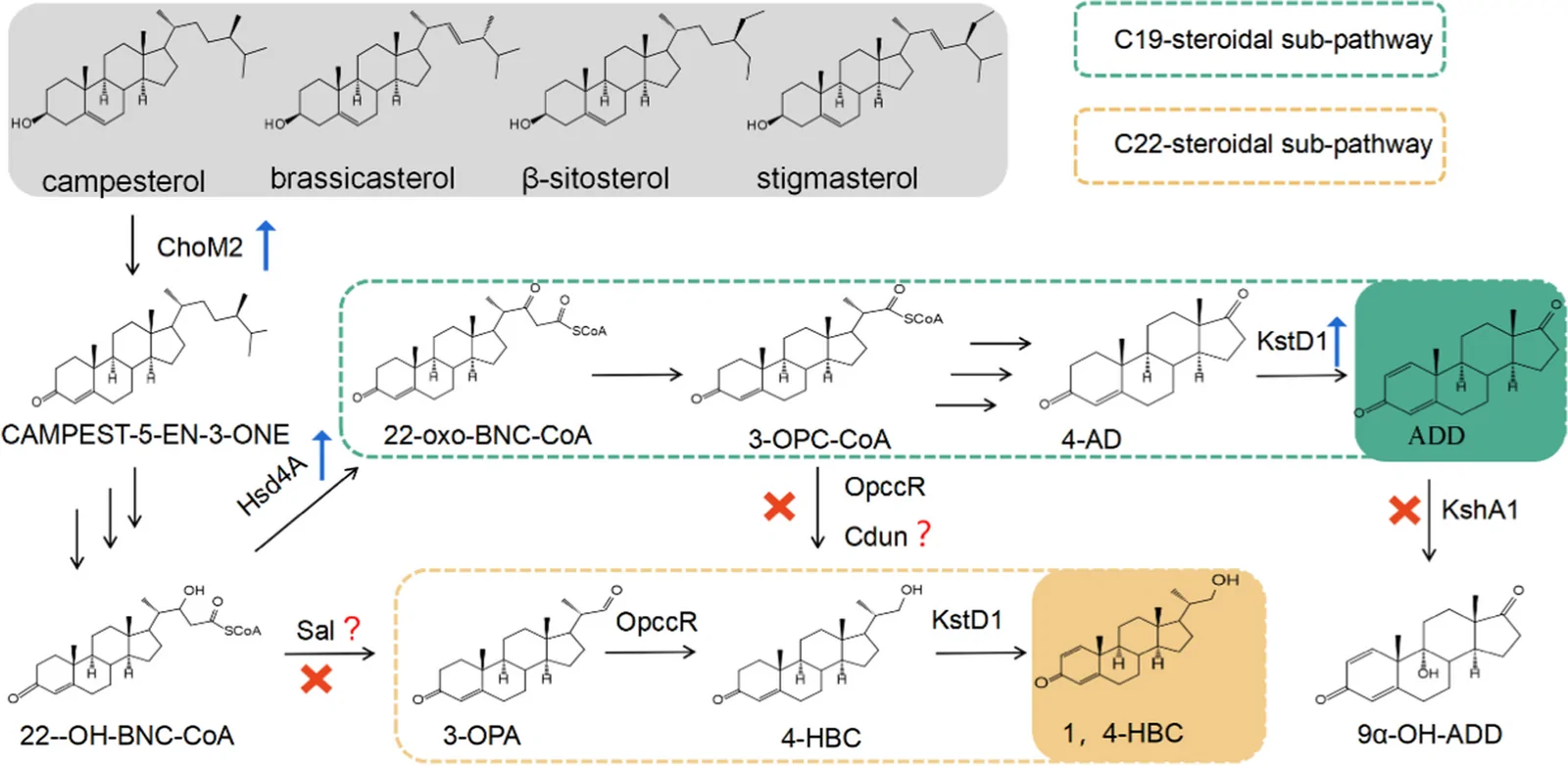
Putative node enzymes in the catabolic switch between the C19- and C22-steroidal subpathways of phytosterol degradation in *M. neoaurum* NRRL B-3805. Abbreviations: 4-AD, 4-androstene-317-dione; ADD, androst-1,4-diene-317-dione; 3-OPA, 3-oxo-4-pregnene-20-carboxyl aldehyde; 4-HBC, 22-hydroxy-23,24-bisnorchol-4-ene-3-one; 1,4-HBC, 22-hydroxy-23,24-bisnorchol-1,4-diene-3-one; ChoM, cholesterol oxidase; Hsd4A, 17-hydroxysteroid dehydrogenase; KstD, 3-ketosteroid-Δ1-dehydrogenase; OpccR, dual-role reductase; Sal, thiolase-like protein; Cdun, short-chain dehydrogenase

There are typically two subpathways involved in the side-chain degradation of phytosterol in *Mycobacterium* (Fig. 1). In addition to the C19-steroid, the C22-steroidal subpathway commonly accumulates 20-hydroxymethyl pregn-4-ene-3-one (4-HBC) and 20-hydroxymethyl pregn-1,4-diene-3-one (1,4-HBC). Some key enzymes are reported to be involved in regulating the metabolic flux distribution between the two subpathways. The 3-ketoacyl-CoA thiolase FadA5 is responsible for the initial step of phytosterol side-chain degradation (Donova and Egorova 2012; Schaefer et al. 2015). Hydroxyacyl-CoA dehydrogenase (Hsd4A) catalyzes the dehydrogenation of 22-hydroxy-3-oxo-cholest-4-ene-24-carboxyl-CoA (22-OH-BNC-CoA) in the second cycle of cholesterol side-chain degradation (Xu et al. 2016). A dual-role reductase (OpccR) was recently reported to catalyze the reduction of 3-oxo-4-pregnene-20-carboxyl-CoA (3-OPC-CoA) and 3-oxo-4-pregnene-20-carboxyl aldehyde (3-OPA) in the production of 4-HBC (Peng et al. 2021). Much attention has been paid to the analysis of metabolic pathways in growing cells (Zhao et al. 2021), while few studies addressed the pathway alterations in resting cells, which are often used as biocatalysts. Clarification of the regulation mechanism underlying the catabolic flow of phytosterol degradation during biotransformation with resting cells is therefore of significant importance, and key node enzymes that may drive the metabolic switch should be considered first. This information may offer insightful ideas for the rational development of mycolicibacterial cell factories to exclusively attain specific products via directed bypass pathway inhibition.

In addition, byproduct accumulation can also be the result of the insufficient activity of rate-limiting enzymes. To develop genetic tools for optimizing biosynthetic pathways, plasmid or genome alterations always require either an adjustment of gene expression or an introduction of heterologous genes, which are regulated by specific promoters and ribosome binding sites (RBS) (Balzer et al. 2013; Englund et al. 2016). Hong et al. constructed a library of 49 ribosomal binding sites (RBS) on the mycobacterial plasmid pMV261, obtaining 38 strong and 11 weak RBS (Sun et al. 2020). Compared with the RBS, the available promoters are relatively simpler and more widely available. The constitutive promoter hsp60 is a popular choice for the overexpression of genes to reinforce biosynthetic pathways (Shao et al. 2019). In contrast to other species of *Mycobacterium*, there are no available strong promoters for *M. neoaurum*, which leads to an excessive dependence of heterologous gene expression on multicopy plasmids. However, the resulting metabolic burden will inhibit cell growth, while the lack of suitable antibiotics, especially for long-term transformation with resting cells, will lead to the loss of plasmids. To achieve stable and effective expression of key rate-limiting enzymes in the phytosterol conversion pathway, it is necessary to mine more suitable and stronger promoters as well as to construct a chromosomal integration platform.

In this study, a two-step biotransformation process was adopted using an ADD-producing strain *M. neoaurum* NRRL B-3805. To investigate the effect of induction with different concentrations of phytosterol on the catabolic switch between the C19- and C22-steroidal subpathways, the expression of putative node enzymes was investigated at the transcriptional level, followed by an analysis of metabolic changes after individual deletion. In addition, promoter engineering as well as overexpression system optimization in *M. neoaurum* NRRL B-3805 was also performed to remove bottlenecks. Finally, directed inhibition of competing pathways and reinforcement of rate-limiting steps resulted in a strong targeted metabolic flux toward ADD synthesis in resting cells of *M. neoaurum* NRRL B-3805, resulting in a high molar yield and volumetric productivity.

## Materials and methods

### Chemicals, strains, and plasmids

The phytosterols (40.50% β-sitosterol, 23.82% campesterol, 24.96% stigmasterol, and 1.07% brassicasterol) were purchased from Cofco Tech Bioengineering Co., Ltd. (Tianjin, China). ADD and 1,4-HBC were obtained from Shanghai Yuanye Bio-Technology Co., Ltd. (Shanghai, China). 2-Hydroxypropyl-β-cyclodextrin (HP-β-CD) was purchased from Sigma-Aldrich Trading Co., Ltd. (Shanghai, China). The DNA polymerase and One-Step Cloning Kit were purchased from Vazyme Biotech Co., Ltd. (Nanjing, China). Restriction enzymes were from Thermo Fisher Scientific (Waltham, Massachusetts, USA). Unless otherwise stated, all other chemicals were obtained from Shanghai Aladdin Bio-Chem Technology Co., LTD, and Sinopharm Chemical Reagent Co., Ltd., China. All strains and plasmids used in this study are listed in Table 1. *M. neoaurum* NRRL B-3805 is a non-pathogenic industrial host deposited at the German National Resource Centre for Biological Material. The other strains were derived from *M. neoaurum* NRRL B-3805 through general genetic manipulation. The promoters and RBS sequences used in this study are listed in Table S1. The plasmids and detailed construction processes were constructed with the primers listed in Supplemental Table S2.

**Table 1:** Strains and plasmids used in this study

Strains	Relevant characteristics	Source
*NB*	*M. neoaurum* NRRL B-3805	NRRL^a^
*NB*_Δ*kshA1*_	NRRL B-3805 *derivative,* Δ*kshA1*	This study
*NB*_Δ*kshA1/opccR*_	NRRL B-3805 *derivative,* Δ*kshA1*Δ*opccR*	This study
*NB*_Δ*kshA1/opccR/cdun*_	NRRL B-3805 *derivative,* Δ*kshA1*Δ*opccR*Δ*cdun*	This study
*NB*_Δ*kshA1/opccR/sal*_	NRRL B-3805 *derivative,* Δ*kshA1*Δ*opccR*Δ*sal*	This study
Plasmids	Relevant characteristics	Source
pMV261	*Mycobacterium* overexpression vector, *Kan*^*R*^	(Yao et al. 2013)
pMV306	*Mycobacterium* integrating vector, *Kan*^*R*^	(Yao et al. 2013)
pMV261-promoter-*kstD1*	pMV261, containing the *kstD1* gene with the indicated promoter	This study
pMV261-promoter-*choM2*	pMV261, containing the *choM2* gene with the indicated promoter	This study
pMV261-promoter-*eGFP*	pMV261, containing the *eGFP* gene with the indicated promoter	This study
pMV306-promoter-*eGFP*	pMV306, containing the *eGFP* gene with the indicated promoter	This study
pMV306-L2-*choM2*	pMV306, containing the *choM2* gene with the L2 promoter for chromosomal integration	This study
pMV306-L2-*kstD1*	pMV306, containing the *kstD1* gene with the L2 promoter for chromosomal integration	This study
pMV306-L2-*hsd4A*	pMV306, containing the *hsd4A* gene with the L2 promoter for chromosomal integration	This study

^a^*NRRL* Agricultural Research Service Culture Collection (Northern Regional Research Laboratory)

### Identification of the OpccR isozymes Cdun and aldolase Sal

The sequences of short-chain dehydrogenase Cdun (AMO04762.1) as OpccR putative isozymes and Sal aldolase (CP011022.1) from *M. neoaurum* NRRL B-3805 were, respectively, performed by amino acid sequence alignments with reported OpccR (QPP46742.1) and Sal (WP_039606914.1) by ClustalX 1.81 software and are listed in Supplementary Material.

### Genetic manipulation

DNA was cloned and purified using standard protocols. Overexpression vectors were constructed by cloning PCR product between the *Bam*HI and *Hin*dIII sites of the shuttle plasmid pMV261 or the *Xba*I and *Hpa*I sites of the shuttle plasmid pMV306. The ClonExpress® II One Step Cloning Kit (Vazyme, China) was used for ligated fragments and linearized vectors in *E. coli* DH5α, and all plasmids were purified from *E. coli* DH5α and verified by DNA sequencing. The overexpression and integration plasmids were electroporated into the competent mycobacterial cells, and positive transformants were further confirmed by PCR and sequencing.

Targeted genetic deletion in *M. neoaurum* was performed as described before (Parish and Stoker 2000; Yao et al. 2013). The P2NIL suicide vector was constructed by inserting the PCR product between the *Pac*I and *Not*I sites. For unmarked gene deletion via homologous recombination in *M. neoaurum*, the plasmid was first subjected to alkali treatment and UV irradiation and then transferred into the mycobacterial cells by electroporation. After a two-step selection process on kanamycin and sucrose plates, the recombinant strains were confirmed by PCR and sequencing. All the primers used are listed in Supplementary Material.

### Quantitative real-time PCR

The RNA was isolated using a FastPure Cell/Tissue Total RNA Isolation Kit, and residual DNA in the sample was removed according to the instructions (Q711, Vazyme, China). Reverse transcription was conducted to obtain cDNA, and qPCR was performed using the ChamQ Universal SYBR qPCR Master Mix. Gene expression was analyzed using the 2^−ΔΔCT^ method (Zhang et al. 2018) with 16S RNA as the internal reference for normalization. Primers were designed online using sequences from the NCBI database and are listed in Supplementary Material.

### Measuring signal intensities of recombinant fluorescent strains

Recombinant strains based on NRRL B-3805 expressing the eGFP constructs were growing for 60 h in LB medium with kanamycin. Then, the cells from 1 mL of the bacterial culture were harvested by centrifugation at 2000 g for 1 min, washed with 1 mL of 10 mM K_2_HPO_4_ buffer (pH = 7.4) three times, and resuspended in the same buffer. Then, 200 µL aliquots were transferred into a clear-bottomed black 96-well plate (Costar) and the plate was placed in a multifunctional microplate reader (BioTek Synergy H1). Fluorescence was measured at an excitation wavelength of 488 nm and emission wavelength of 528 nm. The fluorescence values were normalized to the respective optical density at 600 nm (OD_600_) of the cell suspension in each well.

### Culture conditions and two-step biotransformation processing

For the preparation of resting cells, the strain was first cultivated in LB medium (50 mL in 250-mL shake flasks) at 30 °C and 180 rpm for 36 h and then transferred to 800-mL M3-Tw medium (KH_2_PO_4_ 0.5 g/L, K_2_HPO_4_ 0.5 g/L, (NH_4_)_2_HPO_4_ 1.5 g/L, FeSO_4_·7H_2_O 0.005 g/L, ZnSO_4_·7H_2_O 0.002 g/L, MgSO_4_·7H_2_O 0.2 g/L, yeast extract 5 g/L, and glycerol 5 g/L, adjust pH to 7.2, Tween 80 0.05% (v/v)) in 2-L shake flasks with a 2% inoculum size for the main culture (Loraine and Smith 2017). Phytosterol (0, 0.2, 0.5, 1.0 g/L) was added into the M3-Tw medium as an inducer. After 48 h of incubation at 30 °C and 180 rpm, cells were harvested by centrifugation at 2000 g and 4 °C for 10 min. The cell pellets were washed twice with 20 mM Na^+^-phosphate buffer (pH 8.0) and resuspended in the same buffer to yield the resting cells for further use.

For the biotransformation of phytosterols using the resting cells, the sterol emulsion (100 g/L) was homogenized with HP-β-CD (400 g/L) using dispersion equipment. The bioconversion was performed in a reaction system containing 50 g/L wet cells in 50 mL 20 mM K_2_HPO_4_ (pH 8.0) at 30 °C and 180 rpm in a 250-mL shake flask.

### Analytical methods

Products were analyzed by high-performance liquid chromatography on a G7115A instrument (Agilent Technologies, American) equipped with a C18 column (250 mm × 4.6 mm, 5 μm; J&K Scientific, China). Methanol and water (80:20, v/v) was used as the mobile phase at 1 mL/min, and a UV detector at 254 nm, 40 °C, was used to detect intermediates.

### Statistical analysis

All experiments were performed in triplicates, and the mean values and standard curves calculated from three different batches of data were used in the manuscript. The least significant difference was computed at *p* < 0.05. The figures were performed using the Origin software version 8.0 (OriginLab Corp., Northampton, MA, USA).

## Results

### Induction of phytosterol caused a catabolic switch from C22- to C19-steroidal side-chain cleavage in resting cells of NRRL B-3805_ΔkshA1_

**Fig. 2:**
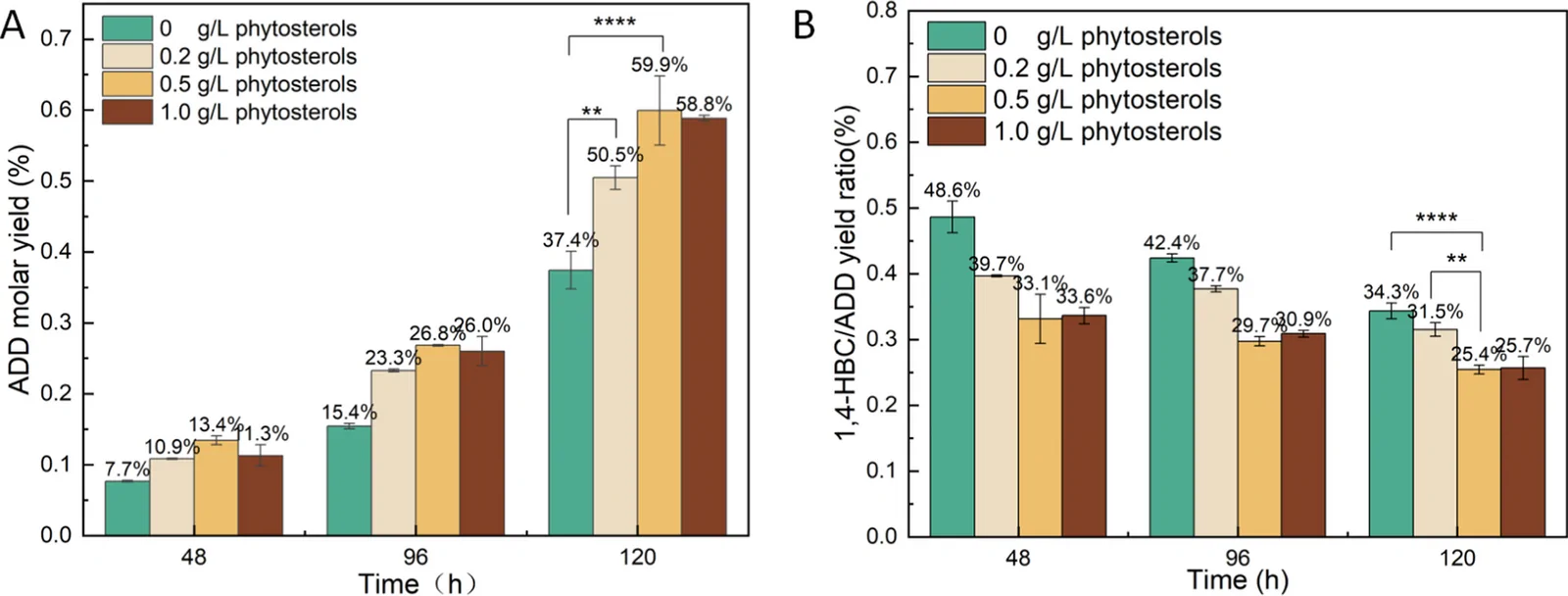
Induction with different concentrations of phytosterol during the cell growth step resulted in an increased ADD accumulation (**A**) instead of 1,4-HBC (**B**). Resting cells of *M. neoaurum* NRRL B-3805_Δ*kshA1*_ were collected as biocatalysts after induction with different concentrations of phytosterol and the biotransformation of phytosterol (10 g/L) was performed. Data are representative of at least three independent experiments; the error bars indicate standard deviation. ****p < 0.0001, **p < 0.01

The enzyme activity of 3-ketosteroid-9α-hydroxylases was disrupted by *kshA1* deletion to obtain the ADD-producing strain *NB*_Δ*kshA1*_. There is an accumulation trend of ADD over time in the biotransformation system, utilizing resting cells of NRRL B-3805 as catalysts, both with and without the deletion of *kshA1* (Fig. S1). The main product was ADD and 1,4-HBC, presenting comparatively high dehydrogenase activity of C1,2 in NRRL B-3805. To investigate the effect of phytosterol induction on the catabolic switch within the resting cells of *NB*_Δ*kshA1*_, different concentrations of phytosterol (0, 0.2, 0.5, 1.0 g/L) were supplemented into the growth medium as inducer, and the accumulation of both ADD and 1,4-HBC was determined during the biotransformation stage. Compared with the negative control, the molar yield of ADD was increased by 37.4 to 50.5%, as well as 59.9% and 58.8% in the groups induced with 0.2, 0.5, and 1.0 g/L phytosterol, respectively (Fig. 2A). On the other hand, although the accumulation of 1,4-HBC gradually increased during the biotransformation process, a significant reduction in the conversion ratio of 1,4-HBC toward ADD was observed with the increased addition of phytosterol (Fig. 2B). These results indicated that proper addition of phytosterol during the cell growth stage rerouted the catabolic flux into the ADD subpathway, indicating that the corresponding enzyme activities in the C19- and C22-steroidal subpathways may be affected by phytosterol even in the early stage for biomass accumulation. Thus, the molecular mechanism underlining the metabolic switch of phytosterol degradation was further explored.

**Fig. 3:**
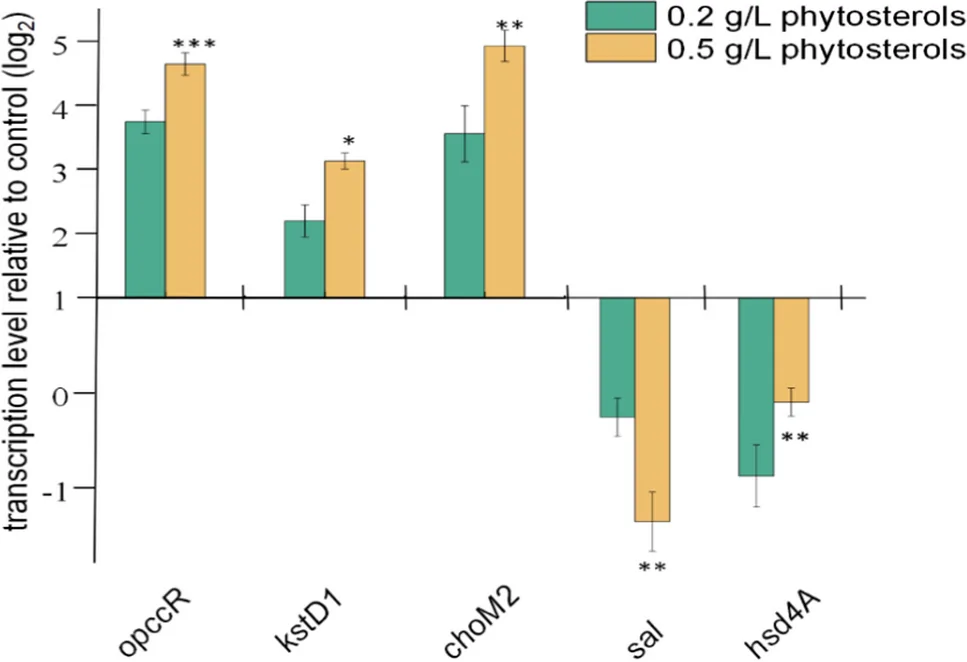
Fold changes in the transcriptional levels of genes related to phytosterol degradation in *M. neoaurum* NRRL B-3805_Δ*kshA1*_ after 24 h of induction with different concentrations of phytosterol. Fluorescence intensity was normalized to the negative control group without induction with phytosterol. Data are representative of at least three independent experiments, and the error bars indicate standard deviations. ****p < 0.0001, **p < 0.01, *p < 0.05

To confirm the above assumption, transcriptional changes of the genes encoding key enzymes involved in phytosterol catabolism were investigated. Cholesterol oxidases (ChoM2) and 3-ketosteroid-1,2-dehydrogenase (KstD1), respectively, play roles in the initiation of phytosterol degradation and introduction of a C1-C2 double bond in 4-HBC or AD (Rohman et al. 2013; Yao et al. 2013). In addition, the bifunctional protein of both OpccR and Hsd4A, important for the redox reaction of C17 sidechain degradation, was also investigated (Peng et al. 2021; Xu et al. 2016). Recently, the Sal aldolase was confirmed to play a role in side-chain degradation of sterols in *Pseudomonas* sp. Chol1 (Holert et al. 2013). Further sequence alignment revealed a putative aldolase sequence in the genome of *M. neoaurum* NRRL B-3805 that shared 58.88% similarity with a homologous Sal (Fig. S3). Aldolases, which belong to the thiolase superfamily, are also essential for the cleavage of the sterol side chain (Holert et al. 2013). The real-time PCR results demonstrated that the transcription of *kstD1 choM2* and *opccR* was sharply upregulated following induction with increased concentrations of phytosterol, while the *sal* gene was downregulated. The transcriptional level of *hsd4A* did not exhibit a regular change, indicating that it was not regulated by phytosterol (Fig. 3). The significant upregulation in the transcriptional levels of *kstD1* and *choM2* reflected the strengthening of the overall metabolic flux in steroid degradation, which was consistent with the increased production of both ADD and 1,4-HBC (Fig. 2A). Moreover, the induction of phytosterol with increased concentrations diverted the end product from 1,4-HBC to ADD, indicating that some key steps might be affected that are involved in the metabolic switch between the C19- and C22-steroidal subpathways. Our data revealed an obvious dose-dependent downregulation of *sal*, which was not consistent with the change trend of *hsd4A* (Fig. 3). Notably, the decline of the 1,4-HBC/ADD yield ratio did not coincide with the downregulation of *opccR* transcription. We speculate that the downregulation of *sal* led to a reduced supply of the precursor 3-OPA, thus affecting the synthesis of 1,4-HBC. In addition, the expression of related enzymes in the biosynthetic pathway of ADD utilizing 3-OPC-CoA as the common precursor was more significantly upregulated than that of *opccR*.

### Blocking of C22-steroidal subpathway by sequential deletion of opccR and sal

It was reported that OpccR plays an important role in the synthesis of 4-HBC (Peng et al. 2021). To block the C22-steroidal subpathway, *opccR* was deleted by homologous recombination in the chassis strain *NB*_Δ*kshA1*_, resulting in *NB*_Δ*kshA1/opccR*_. After deletion of *opccR*, the yield of ADD increased marginally, from 3.87 to 4.08 g/L. At the same time, the yield of 1,4-HBC decreased from 1.58 to 1.36 g/L after 120 h of biotransformation (Table 2). Although the byproduct titer of 1,4-HBC decreased to some extent, it was still present in the bioconversion system. Based on this result, we speculated that isozymes of OpccR may exist in the genome of NRRL B-3805. As a bifunctional enzyme, putative isozymes corresponding to either the C- or N-terminal domain of OpccR were, respectively, screened using the two virtual probes AGRSIR and DGPY, which were, respectively, designed according to conserved sequences found in the C- and N-terminal domain of OpccR. A total of 26 putative isozymes corresponding to the C-terminal domain mainly designated as “short-chain dehydrogenase” were obtained, named as Cdun, which exhibited the highest similarity of 39.2% (Fig. S2), chosen for further deletion. Unfortunately, 1,4-HBC was still present in the phytosterol biotransformation system using resting cells of *NB*_Δ*kshA1/opccR/cdun*_.

**Table 2:** Accumulationof ADD and byproducts in biotransformation using engineered *M. neoaurum* NRRL B-3805 strains with sequential genetic deletions at the indicated time

	Product (g/L)					
	ADD		AD		1,4-HBC	
	Time (h)					
	48	120	48	120	48	120
Strain						
* NB*	2.27 ± 0.23	3.39 ± 0.12	0.15 ± 0.01	0.11 ± 0.01	0.76 ± 0.03	1.66 ± 0.07
* NB*_Δ*kshA1*_	2.35 ± 0.10	3.87 ± 0.13	0.16 ± 0.01	0.13 ± 0.01	0.96 ± 0.04	1.58 ± 0.03
* NB*_Δ*kshA1/opccR*_	2.34 ± 0.13	4.08 ± 0.09	0.16 ± 0.02	0.14 ± 0.01	0.78 ± 0.03	1.36 ± 0.06
* NB*_Δ*kshA1/opccR/Cdun*_	2.36 ± 0.15	4.03 ± 0.11	0.15 ± 0.02	0.12 ± 0.01	0.78 ± 0.01	1.32 ± 0.08
* NB*_Δ*kshA1/opccR/sal*_	3.46 ± 0.25	4.90 ± 0.12	0.23 ± 0.02	0.16 ± 0.02	0.00	0.00

**Fig. 4:**
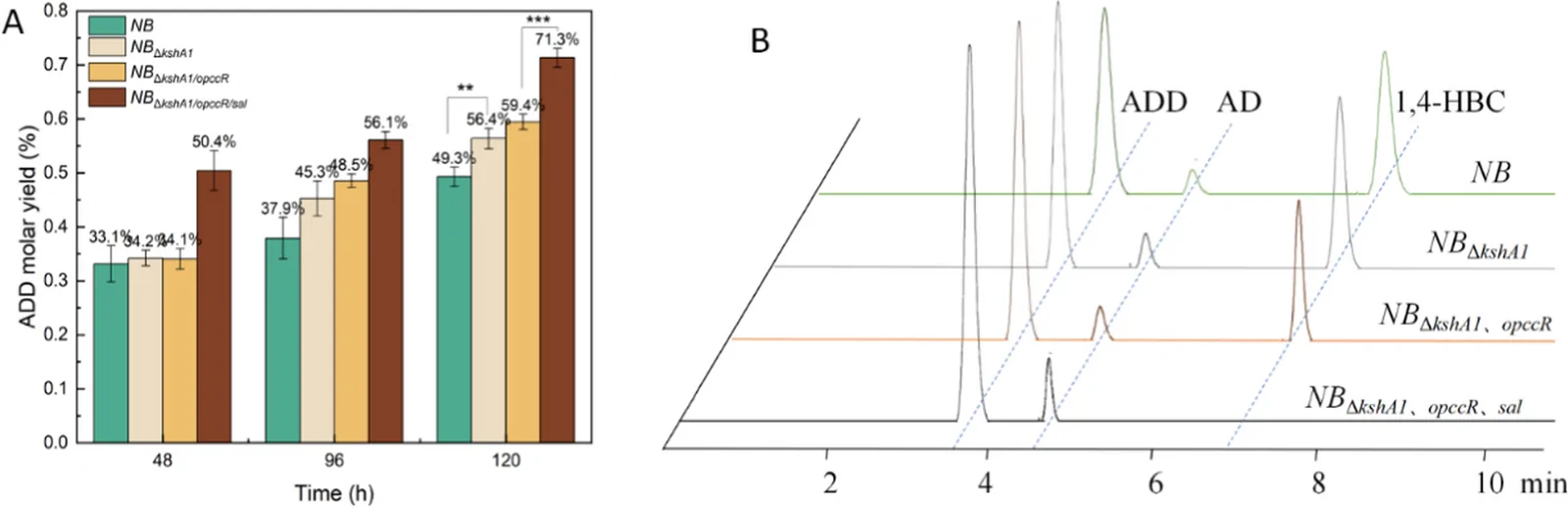
Metabolites accumulated during phytosterol degradation by *M. neoaurum* NRRL B-3805 with sequential deletion of targeted genes. **A** Molar yields of ADD from 10 g/L phytosterol. **B** HPLC chromatogram of steroidal intermediates, mainly including ADD, AD, and 1,4-HBC at 3.81 min, 4.48 min, and 6.73 min, respectively

At the same time, the putative aldolase Sal with a role in the preceding step of C22 steroidal degradation exhibited a dose-dependent downregulation during ADD bioconversion. Thus, the *sal* gene was tentatively deleted from the genome of the *NB*_Δ*kshA1/opccR*_ strain to generate the strain *NB*_Δ*kshA1/opccR/sal*_, which no longer produced the byproduct of 1,4-HBC (Fig. 4B), resulting in a significant increase of ADD accumulation. After 120 h of biotransformation, the ADD yield was increased to 4.90 g/L (Table 2), corresponding to a molar yield of 71.3%, which was 70.3% higher than that of the *NB*_Δ*kshA1/opccR*_ strain (Fig. 4A). This result revealed that the sequential deletion of *opccR* and *sal* led to a complete blockage of the C22-steroidal subpathway. Elimination of the C22-steroidal byproduct in C19-steroidal producing strain was recognized as a great challenge in previous studies (Chang et al. 2020; Liu et al. 2018; Wang et al. 2019; Xu et al. 2015), which greatly hampered the downstream processing. This could be due to the incompletely defined catabolism responsible for the truncation of the sterol C17 side chain, which involves a series of reactions analogous to the fatty acid β-oxidation pathway. Our data confirmed that the putative aldolase Sal plays a crucial role in driving the metabolic switch between the C19- and C22-steroidal subpathways. However, due to the difficulties in obtaining the substrate 22-OH-BNC-CoA for in vitro functional characterization, the precise role of Sal in the degradation of the C24 steroidal side chain needs further confirmation.

### Characterization and overexpression of rate-limiting step in ADD bioconversion via promoter engineering and genome integration

**Fig. 5:**
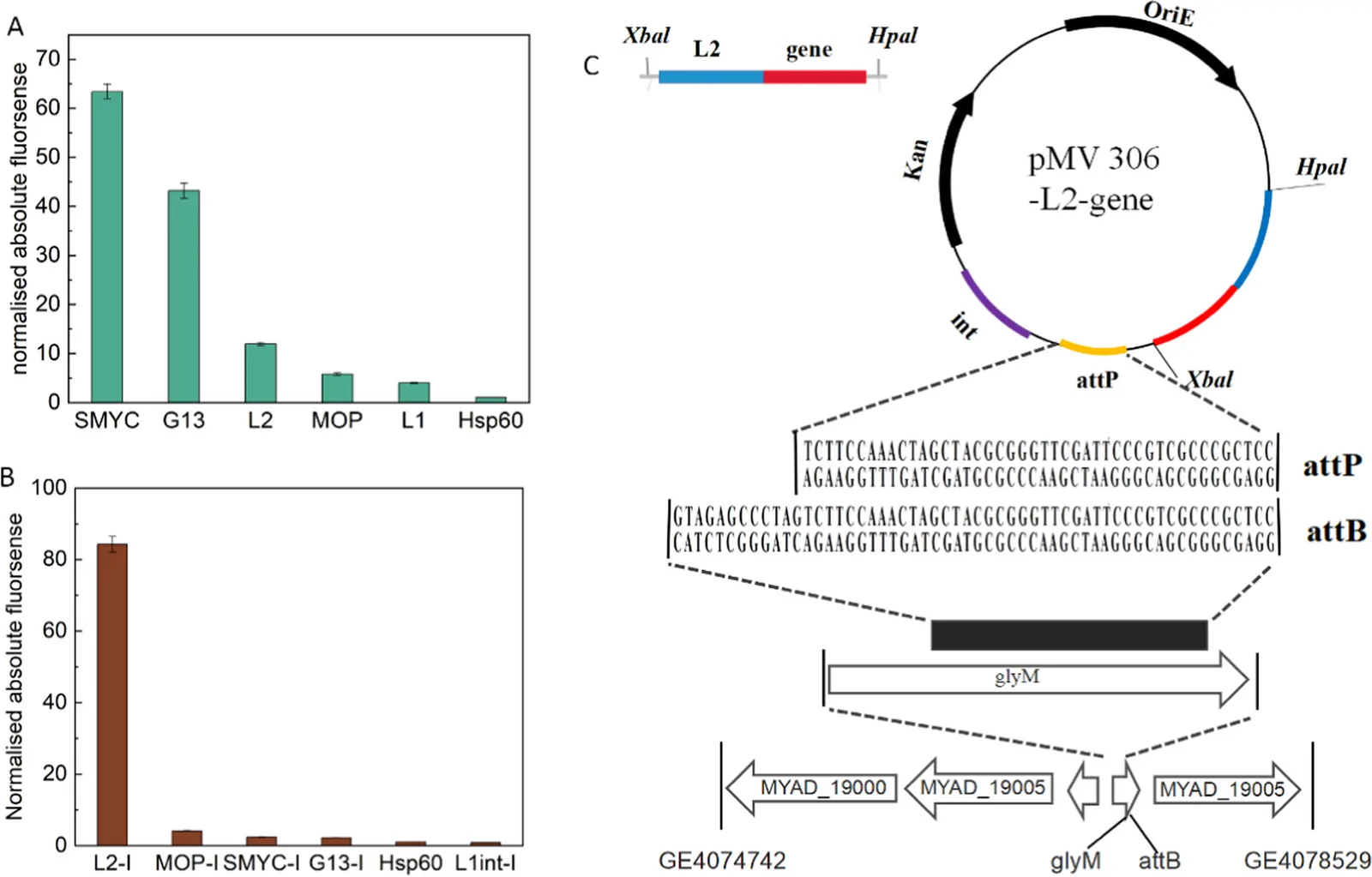
Promoter activity was measured by the eGFP fluorescent intensity and was normalized to the optical density at 600 nm (OD_600_). Mycobacterial cells harboring the variants of promoter-reporter constructs on the pMV261 (**A**) or pMV306 (**B**) backbone were collected for the promoter activity assay. **C** Construction of pMV306 plasmid and integration into the chromosome at the *attB* attachment site (4077232_4077276) overlapping the *glyM* tRNA gene. Homologous fragments (44 bp) at *attP* and *attB* sites were inserted into the genome with the help of integrase

As there was still a small amount of AD in the biotransformation system using resting cells of *NB*_Δ*kshA1/opccR/sal*_ (Fig. 4B), we further investigated the pivotal elements in the bioconversion from AD to ADD. To develop genetic tools for optimizing biosynthetic pathways, we sought to screen a series of promoters by comparing their GFP fluorescent intensity. Previous studies found a variety of novel promoters (SMYC, G13, msp12, MOP, left, left*) showed high transcriptional activity in *Mycobacterium tuberculosis* (Kolbe et al. 2020). However, only the constitutive promoter hsp60 was adopted for overexpression in *M. neoaurum* in previous studies. Thus, the integrative vector pMV 306 and episomal plasmid pMV 261 were used as backbones to construct expression cassettes encoding the *eGFP* gene driven by the SMYC, G13, L2, MOP, L1, or hsp60 promoter. As shown in Fig. 5A, all recombinant strains expressing eGFP showed a higher fluorescence intensity than the reference strain *NB*_phsp60*-eGFP*_. The strongest fluorescent intensity was observed in *NB*_pSMYC*-eGFP*_, with a 63-fold increase above that of *NB*_phsp60*-eGFP*_. In addition, electro-transformation with the vectors based on pMV 306 was used to integrate different promoters into the chromosome by site-specific recombination (Fig. 5C). The highest expression level was observed in *NB*_pL2*-*I*-eGFP*_ with an 83-fold increase above that of *NB*_phsp60*-eGFP*_. By contrast, the other recombinant strains including *NB*_pMOP*-*I*-eGFP*_, *NB*_pSMYC*-*I*-eGFP*_, *NB*_pG13-I*-eGFP*_, and *NB*_pl1-I *-eGFP*_ had similar expression levels to those of *NB*_phsp60*-eGFP*_ (Fig. 5). Based on the comparison of fluorescence intensity, pMV261 with SYMC and G13 MOP promoters as well as pMV306 with the L2 promoter were selected for the subsequent overexpression of key genes.

To further strengthen the directed synthesis of ADD, putative rate-limiting enzymes were respectively overexpressed using different promoters in *NB*_Δ*kshA1-opccR-sal*_, and a higher phytosterol concentration of 30 g/L was used in the resting cell biotransformation systems. According to the transcriptomic analysis, both *kstD1* and *choM2* play important roles in the directed bioconversion of ADD (Fig. 3). As shown in Table 3, after 144 h, the ADD yield of *NB*_Δ*kshA1/opccR/sal-*phsp60*-kstD1*_, *NB*_Δ*kshA1/opccR/sall-*pSMYC*-kstD1*_, and *NB*_Δ*kshA1/opccR/sal-*pL2*-*I*-kstD1*_ reached 13.88, 16.12, and 16.59 g/L, representing increases of 3.73%, 20.48%, and 24.00% compared with *NB*_Δ*kshA1/opccR/sal*_, respectively. Moreover, the byproduct yield of AD was also significantly decreased (Table 3). These results demonstrated that overexpression of the *kstD1* gene was highly effective for overproducing ADD in *NB*_Δ*kshA1/opccR/sal*_. Unfortunately, increasing the copy number of *choM2* in NB-ADD, irrespective of the promoter, did not lead to an increase of the ADD yield compared with *M. neoaurum* NRRL B-3805. Similar results were also obtained with the strain overexpressing *hsd4A* inserted into the chromosome driven by the promoter L2 (data not shown). This may be due to the sufficient endogenous ChoM2 and Hsd4A for ADD biosynthesis after the strain was induced with 0.5 g/L phytosterol. Unexpectedly, although the fluorescence intensity of strains with MOP and G13 on the pMV261 vector was higher than that of the negative control, the bioconversion ratio was not consistent (Table 3), possibly due to the incompatibility between the corresponding promoter and the host *M. neoaurum* NRRL B-3805.

**Table 3:** Individual overexpression of *kstD1* and *choM2* using selected promoters in strain *NB*_Δ*kshA1/opccR/sal*_ for increased conversion of AD into ADD

	(g/L)			
	ADD		AD	
	Enzyme			
	KstD1	ChoM2	KstD1	ChoM2
Promoter				
hsp60	13.88 ± 0.41	12.38 ± 0.70	1.67 ± 0.03	1.79 ± 0.07
SMYC	16.12 ± 1.04	11.99 ± 1.25	0.91 ± 0.20	1.69 ± 0.04
G13	12.68 ± 0.88	12.86 ± 0.57	1.59 ± 0.05	1.59 ± 0.07
MOP	11.05 ± 1.21	11.56 ± 1.13	1.49 ± 0.05	1.69 ± 0.03
L2-I	16.59 ± 0.33	13.07 ± 0.57	0.78 ± 0.07	1.60 ± 0.05

### Directed biotransformation from phytosterol to ADD by resting cells of engineered *M. neoaurum* NRRL B-3805

**Fig. 6:**
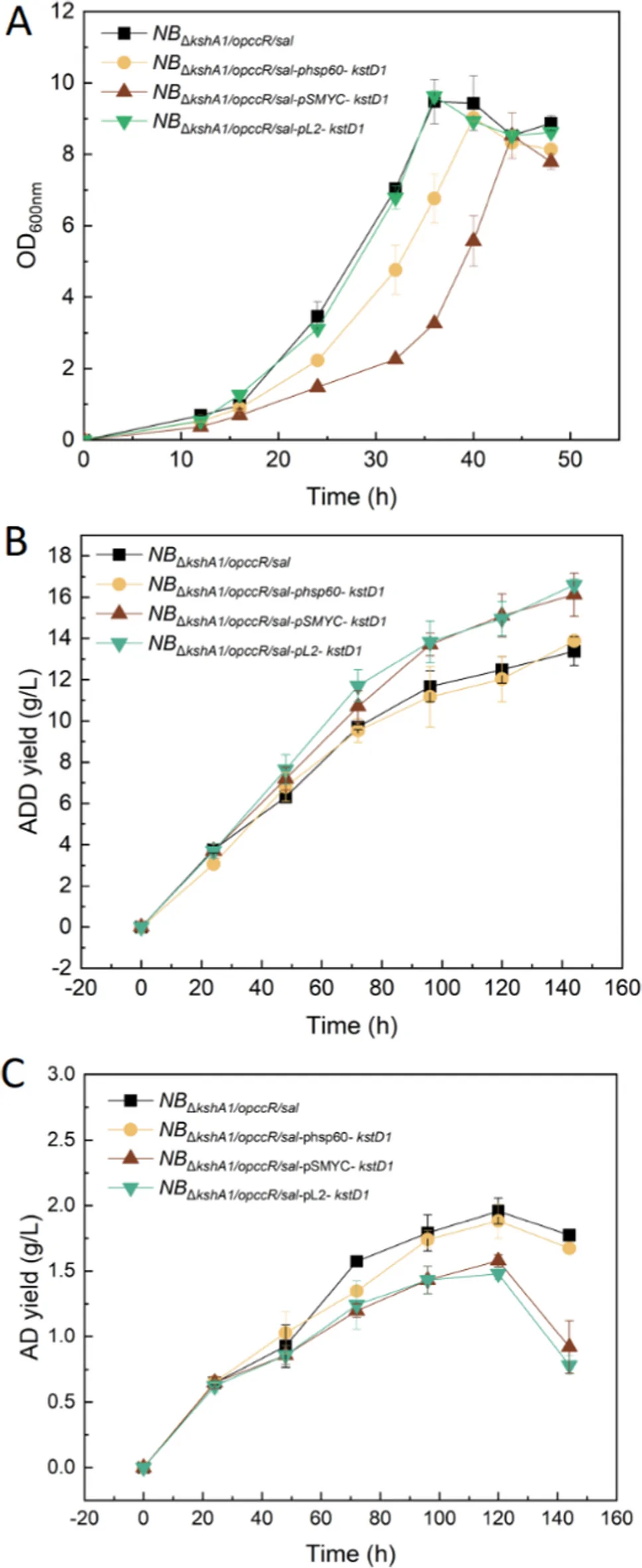
Time course of the profiles of (**A**) OD_600_, (**B**) ADD, and (**C**) AD concentrations during biotransformation of phytosterols (30 g/L) by resting cells of engineered *M. neoaurum* NRRL B-3805_Δ*kshA1-opccR-sal-pL2-kstD1*_ via a two-step bioconversion processing

For the efficient bioconversion of highly concentrated phytosterols into ADD, a two-step bioprocess was adopted. In the first step for the preparation of resting cells, the biomass and initial growth rate were significantly reduced in recombinant strains with plasmid-based gene overexpression (*NB*_Δ*kshA1*/*opccR*/*sal*-pSMYC-kstD1_ and *NB*_Δ*kshA1*/*opccR*/*sal*-phsp60-kstD1_) compared with the negative control. For example, *NB*_Δ*kshA1*/*opccR*/*sal*-pL2- kstD1_ remained to have reached the highest OD_600_ of 9.6 after 36 h of cultivation (Fig. 6A). To overcome growth inhibition due to the metabolic burden of multicopy plasmid expression, chromosomal integration of target genes driven by a strong promoter such as L2 was adopted for the overexpression of rate-limiting enzymes in this study. For the subsequent biotransformation, resting cells were collected and used as the catalyst. As shown in Fig. 6B, the titer of ADD increased dramatically during the biotransformation. In the period of 120 to 144 h, the AD titer sharply decreased and was especially low in strains *NB*_Δ*kshA1/opccR/sal-*pSMYC*-kstD1*_ and *NB*_Δ*kshA1/opccR/sal-*pL2*-*I*-kstD1*_ (0.91 and 0.78 g/L), representing respective 48.6% and 56.0% reductions compared with *NB*_Δ*kshA1/opccR/sal*_. Ultimately, the highest ADD molar yield of 80.5% was obtained with the *NB*_Δ*kshA1/opccR/sal-*pL2*-*I*-kstD1*_ strain, combined with a space–time productivity of 2.77 g·L^−1^·day^−1^. In the subsequent optimization process, bioconversion parameters including the dissolved oxygen and biocatalyst loading as well as substrate concentration on the molar conversion ratio of phytosterol to ADD were, respectively, investigated as shown in Supplementary Material. Under the conditions of 80 g/L wet cell sediment within a liquid volume of 30 mL, the molar biotransformation rate of ADD reached 82.0% and the space–time yield increased from 2.77 to 4.22 g·L^−1^·day^−1^ (Fig. S4).

## Discussion

The cholesterol biodegradation is primarily regulated by two transcriptional repressors from the TetR family in mycobacteria, known as KstR and KstR2 (Kendall et al. 2010; Ma et al. 2022). Target genes of KstR are involved in cholesterol trans-membrane translocation, side-chain truncation, and degradation of nucleus rings A and B. Meanwhile, KstR2 mainly regulates the expression of genes involved in the degradation of steroid nucleus rings C and D (Zhao et al. 2021). Research on the mechanism of KstR regulation suggests that the endogenous ligands of KstR are thioesters of cholesterol side-chain degradation products, including four steroidal rings and the CoA-esterified side-chain moiety. Ligand binding to KstR relieves its inhibition on DNA binding (Ho et al. 2016). In *Nocardioides simplex* VKM Ac-2033D, induction with phytosterols resulted in the upregulation of 91 genes and the downregulation of 41 genes, with approximately one-third of the operons being induced (Shtratnikova et al. 2021). These results suggest that phytosterol induction may relieve the inhibition of enzyme expression by ligand binding of the intermediate during phytosterol degradation, composing the similar structure with steroidal rings and a CoA-esterified side chain. According to our results, the addition of phytosterols during the growth stage induced the expression of related enzymes, leading to increased production of C-19 steroids and the C-19/C-22 ratio was largely increased during the biotransformation stage. Changes in transcription levels of key genes in the degradation pathway further supported this hypothesis, indicating that phytosterols can regulate the expression of related genes, similar to the mechanism of action of regulatory factors such as TetR on genetic clusters involved in cholesterol degradation.

Key enzymes with putative dominant role in the metabolic switch between C-19 and C-22 pathway were committed in recent studies. The dual-functional reductase OpccR is thought to be involved in two different metabolic branches of the C-22 pathway (Peng et al. 2021). Deletion of *opccR* could eliminate 4-HBC in the *M. neoaurum* CCTCC AB2019054 strain, and overexpression of *opccR* combined with *hsd4A* deletion led to the accumulation of 4-HBC as the sole product from phytosterols. Paradoxically, a most recent study showed that *opccR* deletion in *M. neoaurum* HGMS2 strain promoted the accumulation of the C-22 steroid intermediate 22-hydroxy-23,24-bisnorchol-4-ene-3-one, confirming that different branches of the phytosterol degradation pathway are present in different actinobacteria (Song et al. 2023). In our results, the effect of *opccR* deletion on ADD accumulation was not obvious; the byproducts of C-22 steroids could not be completely eliminated in high-yield C-19 steroid strains in previous studies (Chang et al. 2020; Shao et al. 2019). After the deletion of the new identified aldose Sal, the C-22 subpathway was totally abolished in the present work, supporting the target biotransformation from phytosterol to C-19 steroid, a new strategy for the construction of C-19 steroid high-yield strain.

In order to achieve overexpression of rate-limiting enzymes, episomal gene overexpression is commonly adopted using multicopy of plasmid as expression vectors due to the insufficient activity of the constitutive promoter, such as hsp60. In our study, we excavated and engineered novel promoters L2 with high transcriptional activity, which significantly improved the overexpression of target enzymes in *M. neoaurum*. Additionally, Saviola and Bishai demonstrated the integration of a vector carrying a new attB site into the chromosome of *M. smegmatis*, allowing the plasmid-borne attB site to serve as a second site for subsequent integration (Saviola and Bishai 2004). The new integrative approach combined with the strong promoter L2 as applicated in the present study enabled a substantial enhancement of the rate-limiting enzyme and resulted in significantly higher molar yields in the engineered cell factory. Co-expression of multiple genes for the construction of other bacterial strains via this strategy could be envisioned in future study. The production of steroid drug intermediates can be increased and the utilization efficiency of phytosterol can be improved by enhancing metabolic flux. Overexpression of *choM2* resulted in a 40% increase in ADD production in *M. neoaurum* NwIB-R10 and a 51.2% increase in AD production in *M. neoaurum* NwIB-01MS (Yao et al. 2013). However, possibly due to the strong induction effect of phytosterol in growing cells of *M. neoaurum* NRRL B-3805_Δ*kshA1-opccR-sal*_, the endogenous ChoM was already able to efficiently convert phytosterols; therefore, no further improvement in ADD production was observed after *choM* overexpression. KstD1 plays critical role in the dehydrogenation from AD to ADD. In *M. neoaurum* ATCC 25795_△*kshA1*/*kshA2*_, the accumulated AD and ADD from phytosterols could be enhanced by overexpressing *kstD1*, leading to an increased proportion of ADD in the steroid products (Yao et al. 2014). Similarly, in *M. neoaurum* JC-12, ADD yield could be further enhanced by deleting *kshA* and overexpressing *kstD* (Shao et al. 2019). Consistently in our study, the ADD production was also increased in *M. neoaurum* NRRL B-3805. On the other hand, unexpectedly, overexpression of *hsd4A* showed significant improvement in the yield of C-19 steroids in previous studies, while it did not yield promising results in our strain. We speculate that this may be due to the disruption of the C-22 pathway after *sal* deletion, and the endogenous induction of *hsd4A* itself is sufficient for metabolic flux switch toward the C-19 steroidal subpathway (Liu et al. 2018; Wang et al. 2019). Therefore, no further improvement in ADD production with *hsd4A* overexpression was observed after *sal* deletion.

To provide a close resemblance of the industrial application scenario, the molar number of substrates was directly calculated by the initial loading weight of phytosterol in the present study. Phytosterols are complex mixtures of various substances with particles of different diameters. Some insoluble substrate was still observed in the system, especially at high substrate loading, thereby affecting the precise calculation of the molar ratio bioconversion from phytosterol to ADD. Mancilla et al. analyze the effect of the particle size in the biotransformation of phytosterols to AD, and the results show that smaller particles would be suitable for biotransformation (Mancilla et al. 2018). Excluding our efforts on the metabolic engineering for directed biotransformation from phytosterol to ADD, several challenges still remained in the modular optimization of the cell factory, including the cytotoxicity of propionyl-CoA and reactive oxygen species within the cells (Sun et al. 2019; Zhou et al. 2019b), the impact of the thick cell wall of mycobacteria on phytosterol uptake (Abuhammad 2017), and the influence of electron transfer rate on phytosterol degradation (Su et al. 2017; Zhou et al. 2019a). Currently, researchers have proposed corresponding strategies. For example, the deficiency of this gene raised the cell permeability, thereby enhancing the steroid uptake and utilization. The 9α-OH-AD yield in the fbpC3-deficient 9α-OH-AD-producing strain was increased by 21.3% (Xiong et al. 2022). By augmenting catalase CAT and enhancing the synthesis of mycothiol and ergothioneine, the cell viability and the 4-HBC production were enhanced by about 50% (Sun et al. 2019). It is envisioned that a combined optimization of multiplex modular in phytosterol bioconversion system should be performed to maximize the molar yield of steroidal intermediate for industrial applications.

In this study, the catabolic switch of phytosterol degradation between the steroidal C19- and C22-subpathways was investigated in resting cells of *M. neoaurum* NRRL B-3805, which revealed a dose-dependent transcriptional response of putative node enzymes. The byproduct 1,4-HBC was eliminated after sequential deletion of *opccR* and *sal*. Rate-limiting steps were identified and overcome by mining and chromosomal integration of novel strong promoters. The directed biotransformation route developed in this study allowed up to 82.0% molar yield of ADD in the engineered strain, while the enzymes responsible for the catabolic switch as well as the genetic tools provide a new effective method for the directed biotransformation of C19- and C22-steroidal products using phytosterol as the raw material.

## Supplementary information

Below is the link to the electronic supplementary material.

Supplementary file1 (PDF 864 KB)

## Data Availability

The datasets generated during and/or analyzed during the current study are available from the corresponding author on reasonable request.
