# Supplementary material for: Rerouting phytosterol degradation pathway for directed androst-1,4-diene-3,17-dione microbial bioconversion
Source: Appl Microbiol Biotechnol. 2024 Feb 1;108:186. doi: 10.1007/s00253-023-12847-z (PMC10834601; doi:10.1007/s00253-023-12847-z)

Supplementary Material

Title

Rerouting phytosterol degradation pathway for directed androst-1,4-diene-3,17-dione microbial bioconversion

Authors and affiliation:

Xia Ke [1, 2], Jia-Hao Cui [1, 2], Qi-Jie Ren [1, 2], Tong Zheng[1, 2], Xin-Xin Wang [1, 2], Yu-Guo Zheng[1, 2], Zhiqiang Liu [1, 2*]

1 National and Local Joint Engineering Research Center for Biomanufacturing of Chiral Chemicals, Zhejiang University of Technology, Hangzhou 310014, People's Republic of China

2 Key Laboratory of Bioorganic Synthesis of Zhejiang Province, College of Biotechnology and Bioengineering, Zhejiang University of Technology, Hangzhou 310014, People's Republic of China

Table S1 Sequence of selected promoter and RBS used for the overexpression of rate-limiting enzymes.

Promoters	RBS	Sequence
SMYC	AGGAGG	GGATCGTCGGCACCGTCACGGCCGTGGGAGGCGGCA<br>CGATCCGCGACGTGATGATCGGCCGCATCCCCACGGT<br>GCTGCGCAGTGAGCTCTACGCCATCCCGGCGTTGATC<br>TGTGCGTTCGCACGCACAGGCCCGGTGTGAGAAGGG<br>TCTCTGACGAGCGGGAGAACCCACCCGGGGTGGGCG<br>AGTTTGTCCTGCGTGTGCTCGGTCGAGTAGGCTCTGG<br>GAGTACCCGTGTGTACGACCAGCACGGCATACATCA<br>TTTCGACGCCGAGAGATTCGCCGCCCGAAATGAGCA<br>CGATCCGCATGCTTAATTAAGGAGGTATCTCCATG
G13	AGGAGG	GATCGCCACTAGCGCCGCGGTCGGAATCAGCGCACT<br>GGCCGCCGGAGCGGTCAGCTACCTGTTGCTGCGCCGC<br>TTCGCTTTCTAGCACCGACGTCCCGGCGCCAGCAGCT<br>ACCTCTCGAGCAAACGTTTTGCGCGCCCGAAAAATA<br>AGTCGTTGCAGAACTTTCATGAATTAGGCCTTGCTGC<br>GCCCAGGCTCCAGTAGTAGAAATGGAGTCACGGCAG<br>CCCGGTGAAGCCAAGGTCGAACCGGAAGAGAAGGTT<br>CGTCCTCCCGACCCGGGCACCCAGCACGGCCCCCGG

		AACCCACGCGGAGTCATAGCCGCGATAATGGCAGAA
		GTGTTGCGGGCCTGCGTAATTGCGAAATTCAGATGGT
		GCCGACGGCCCTTTGGGTGGGGCTGCAGCCAGAAGG
		GTCGCAAAAGCGCCGAGGCCAACCCACGCAGCCACA
		AATGCACGCTTGGTAACCGAAGGAGGTATCTCCATG
MOP	AGGAGG	GGCTCTAGCGCCGATGGTAGTGTGGGGTCTCCCCATG
		CGAGAGTAGGGAACTGCCAGGCATCAAATAAAACGA
		AAGGCTCAGTCGAAAGGCTGGGCCTTTCGTTTTATCT
		GTTGTTTGTCGGTGAACGCTCTCCTGAGTAGGACAAA
		TCCGCCGGGAGCGGATTTGAACGTTGCGAAGCAACG
		GCCCGGAGGGTGGCGGGCAGGACGCCCGCCATAAAC
		TGCCAGGCATCAAATTAAGCAGAAGGCCATCCTGAC
		GGATGGCCTTTTTGCGTTTCTACAAACTCTTCCTGTCG
		TCATATCTAGACCAGGCTTGACACTTTATGCTTCCGG
		CTCGTATAATGTGTGGAATTGTGAGCGCTCACAATTC
		GGATCCAGCGATGTCGACAGGAGGATTCACCATG
L1	AGGGAGA	CGATGATAAGCGGTCAAACATGAGAATTCGCGGCCG
		CATAATACGACTCACTATAGGGATCTTAATTAAGGCG
		CCTCATGTTCTTTCCTGCGTTATCCCCTGATTCTGTGG
		ATAACCGTATTACCGCCTTTGAGTGAGCTGATACCGC
		TCGCCGCAGCCGAACGACCGAGCGCAGCGAGTCAGT
		GAGCGAGGAAGCGGAAGAGCGCCCAATACGCAAAC
		CGCCTCTCCCAGATCTGATATCGCTAGAGGAAACAGC
		TATGACCATGATTACGCCAAGCTTGCATGCCTGCAGC
		TAGGGCACCAATTTGCGATTAGGGCTTGACAGCCACC
		CGGCCAGTAGTGCATTCTTGTGTCACCGCAGCAGCAA
		GGCGGTAGGCGGATCCGAGAGGATCGTGCCGGTGCC
		GGTGAAAATCCGGCGGCAAGATTCTCCGGTTTGACA
		GCCACCCGGTTATCGGGTAAGCTGCAAGCATCACCA
		ACTTGGACGGGAAAGGGAGATCGTCCATG
L2	AGGAGG	CGATGATAAGCGGTCAAACATGAGAATTCGCGGCCG
		CATAATACGACTCACTATAGGGATCTTAATTAAGGCG
		CCTCATGTTCTTTCCTGCGTTATCCCCTGATTCTGTGG
		ATAACCGTATTACCGCCTTTGAGTGAGCTGATACCGC
		TCGCCGCAGCCGAACGACCGAGCGCAGCGAGTCAGT
		GAGCGAGGAAGCGGAAGAGCGCCCAATACGCAAAC
		CGCCTCTCCCAGATCTGATATCGCTAGAGGAAACAGC
		TATGACCATGATTACGCCAAGCTTGCATGCCTGCAGC
		TAGGGCACCAATTTGCGATTAGGGCTTGACAGCCACC
		CGGCCAGTAGTGCATTCTTGTGTCACCGCAGCAGCAA
		GGCGGTAGGCGGATCCGAGAGGATCGTGCCGGTGCC
		GGTGAAGAGAGATTCGCCGCCCGAAATGAGCACGAT
		CCGCATGCTTAATTAAGGAGGTATCTCCATG

Table S2 Primers used in present study

Primers	Sequence	Application
**Genetic deletion**		
A1-U-F	aatgccgatatcttaattaaacgtcgggcatatcgtgatcgtcg	*kshA1-up*
A1-U-R	ggtgggcctcccgtatctggtc	
A1-D-F	ccagatacgggaggcccacctgaccacgcatgacgagcgg	*kshA1-down*
A1-D-R	tgaattggtaccgcggccgcaagaatccgagccacctgttcgc	
pR-U-F	aatgccgatatcttaattaaccgatgcgggcgttgggttc	*opccR-up*
pR-U-R	caggccacacctatcgtggtgacg	
pR-D-F	gtgtggcctgggtcgtctgtgtggcggcgtacg	*opccR-down*
pR-D-R	tgaattggtaccgcggccgcgtcttcgccaccgtgtccgatgg	
un-U-F	aaccgtattcctttaattaaagcacacgcgcccagacc	*cdun-up*
un-U-R	tctctctgcgatttcggcgcagtacgacgcgctgcgcc	
un-D-F	gcgccgaaatcgcagagagaatg	*cdun-down*
un-D-R	tgaattggtaccgcggccgctcctggaaaagcgcgatcccg	
al-U-F	aaccgtattcctttaattaaagttcgacggcgagttcctcg	*sal-up*
al-U-R	gaattgcacccacacctcgtcggtttacgctcccgtcagcc	
al-D-F	acgaggtgtgggtgcaattctcac	*sal-down*
al-D-R	tgaattggtaccgcggccgcagcgttgatcagaaaccagagtctttgc	
**Quantitative-PCR**		
q-*hsd4A*-F	tcaagagcccatgagctcggtgg	*q-hsd4A*
q-*hsd4A*-R	atgaacgacaacccgatcgacctg	
q-*sal*-F	ttgggtttgcgtggtgacgcag	*q-sal*
q-*sal*-R	tcaatccccctccaggacgagag	
q-o*pccR*-F	ctaccagtgcacacccggaatcg	*q-opccR*
q-*opccR*-R	atggcccgcatgcattatgtcg	
q-*choM2*-F	ttgctgacaagacggcggttcc	*q-choM2*
q-*choM2*-R	gatcgcctcgatattgcgttcggc	
q-*kstD1*-F	ggaatctacgttcgcgaggccg	*q-kstD1*

q-*kstD1*-R	gcgctgatacttggtgcgcatct	
q-16s rRNA-F	tcacgaacaacgcgacaaac	*q-16s rRNA*
q-16s rRNA-R	gcggtaatacgtagggtccg	
**Overexpression**		
261-GFP-hsp60-F	caatggccaagacaattgcggatccatggtctcgaagggcgagga	*pMV261-*
261-GFP-hsp60-R	cagtcgatcgtacgctagttaactcacttgtacagctcgtccatgcccag	*hsp60-eGFP*
261-GFP-smyc-F	gtaccagatctttaaatctagaggatcgtcggcaccgtcac	*pMV261-*
261-GFP-smyc-R	cctcgcccttcgagaccatggagatacctccttaattaagcatgcgg	*smyc- eGFP*
261-GFP-g13-F	taccagatctttaaatctagagatcgccactagcgc	*pMV261-*
261-GFP-g13-R	cctcgcccttcgagaccatggagatacctccttcggttaccaagc	*g13- eGFP*
261-GFP-mop-F	taccagatctttaaatctagaggctctagcgccgatggtagt	*pMV261-*
261-GFP-mop-R	cctcgcccttcgagaccatggtgaatcctcctgtcgacatcgc	*mop- eGFP*
261-GFP-L1-F	cggtaccagatctttaaatctagacgatgataagcggtcaaac	*pMV261-L1-*
261-GFP-L1-R	tcctcgcccttcgagaccatggacgatctccctttcc	*eGFP*
261-GFP-L2-F	taccagatctttaaatctagaccgatgataagcggtcaaacatgag	*pMV261-L2-*
261-GFP-L2-R	cctcgcccttcgagaccatggagatacctccttaattaagcatgc	*eGFP*
306-GFP-smyc-F	aacgcgtgcggccgctctagaggatcgtcggcaccgtcac	*pMV306-*
306-GFP-smyc-R	gtcgatcgtacgctagttaactcacttgtacagctcgtccatgccca	*smyc- eGFP*
306-GFP-g13-F	aacgcgtgcggccgctctagagatcgccactagcgccgc	*pMV306-*
306-GFP-g13-R	gtcgatcgtacgctagttaactcacttgtacagctcgtccatgccca	*g13- eGFP*
306-GFP-mop-F	caacgcgtgcggccgctctagaggctctagcgccgatggtagt	*pMV306-*
306-GFP-mop-R	gtcgatcgtacgctagttaactcacttgtacagctcgtccatgccca	*mop- eGFP*
306-GFP-L1-F	aacgcgtgcggccgctctagacgatgataagcggtcaaacatgagaattcg	*pMV306-L1-eGFP*
306-GFP-L1-R	gtcgatcgtacgctagttaactcacttgtacagctcgtccatgccca	
306-GFP-L2-F	aacgcgtgcggccgctctagacgatgataagcggtcaaacatgagaattcg	*pMV306-L2-eGFP*
306-GFP-L2-R	gtcgatcgtacgctagttaactcacttgtacagctcgtccatgccca	
261-choM2-smyc-F	attaaggaggtatctccttgctgacaagacggcggttc	*pMV261-*

261-choM2-smyc-R	cagtcgatcgtacgctagttaacctaacgccggcctgagatgatc	*smyc-choM2*
261-kstD1-smyc-F	attaaggaggtatctccatgactgaacaggactacagtgtctttg	*pMV261-*
261-kstD1-smyc-R	agtcgatcgtacgctagttaactcaggcctttccagcgagatgc	*smyc-kstD1*
261-choM2-hsp60-F	tgcggatccagctgcagaattcatgctgacaagacggcggttcc	*pMV261-*
261-choM2- hsp60-R	acgtcgacatcgataagcttctagatcgcctcgatattgcgttcggc	*hsp60-choM2*
261-kstD1-hsp60-F	cggatccagctgcagaattcgtgtggattccgaacaacgaggttct	*pMV261-*
261-kstD1-hsp60-R	acgtcgacatcgataagctttcaggcctttccagcgagatgca	*hsp60- kstD1*
261-choM2-g13-F	ccgaaggaggtatctccttgctgacaagacggcggttc	*pMV261-*
261-choM2-g13-R	gcagtcgatcgtacgctagttaacctaacgccggcctgagatgatcgc	*g13-choM2*
261-kstD1-g13-F	aaccgaaggaggtatctccatgactgaacaggactacagtgtctttg	*pMV261-*
261-kstD1-g13-R	cagtcgatcgtacgctagttaactcaggcctttccagcgagatgc	*g13-kstD1*
261-choM2-mop-F	cgacaggaggattcaccttgctgacaagacggcggttcct	*pMV261-*
261-choM2-mop-R	cagtcgatcgtacgctagttaacctaacgccggcctgagatgatcgc	*mop-choM2*
261-kstD1-mop-F	gtcgacaggaggattcaccatgactgaacaggactacagtgtctttg	*pMV261-*
261-kstD1-mop-R	cagtcgatcgtacgctagttaactcaggcctttccagcgagatgc	*mop-kstD1*
306-hsd4A-L2-F	attaaggaggtatctccatgaacgacaacccgatcgacctg	*pMV306-L2-*
306-hsd4A-L2-R	gtcgatcgtacgctagttaactcaagagcccatgagctcggtg	*hsd4A*
306-choM2-L2-F	attaaggaggtatctccttgctgacaagacggcggttc	*pMV306-L2-*
306-choM2-L2-R	gtcgatcgtacgctagttaacctaacgccggcctgagatgatc	*choM2*
306-kstD1-L2-F	attaaggaggtatctccatgactgaacaggactacagtgtctttg	*pMV306-L2-*
306-kstD1-L2-R	gtcgatcgtacgctagttaactcaggcctttccagcgagatg	*kstD1*

Figure S1 Time course accumulation of ADD in the biotransformation system using resting cells of *M. neoaurum* NRRL B-3805 as the catalysts with/without *kshA1* deletion.

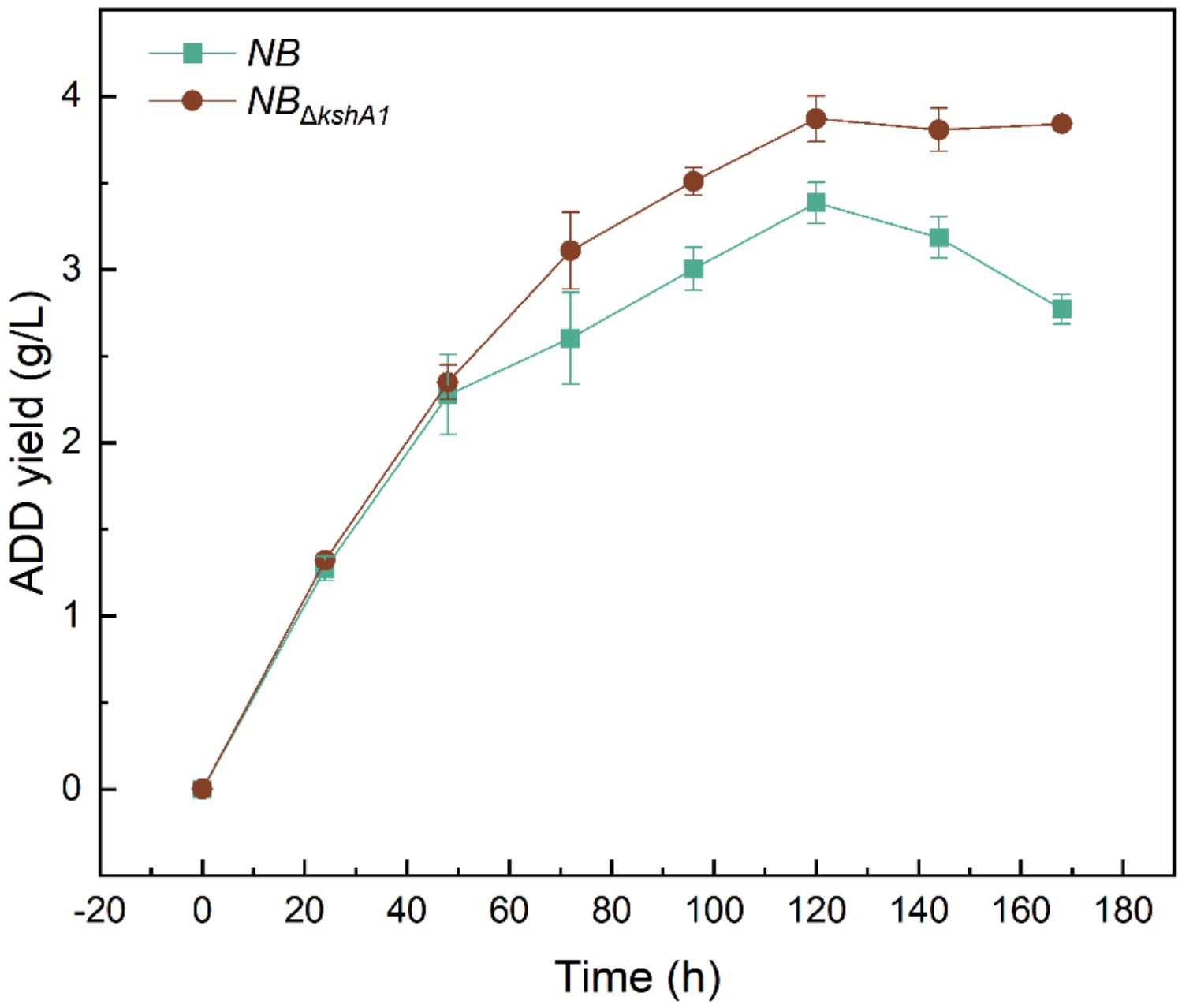

Figure S2 Amino acids sequence alignment between C-terminal of OpccR and Cdun.

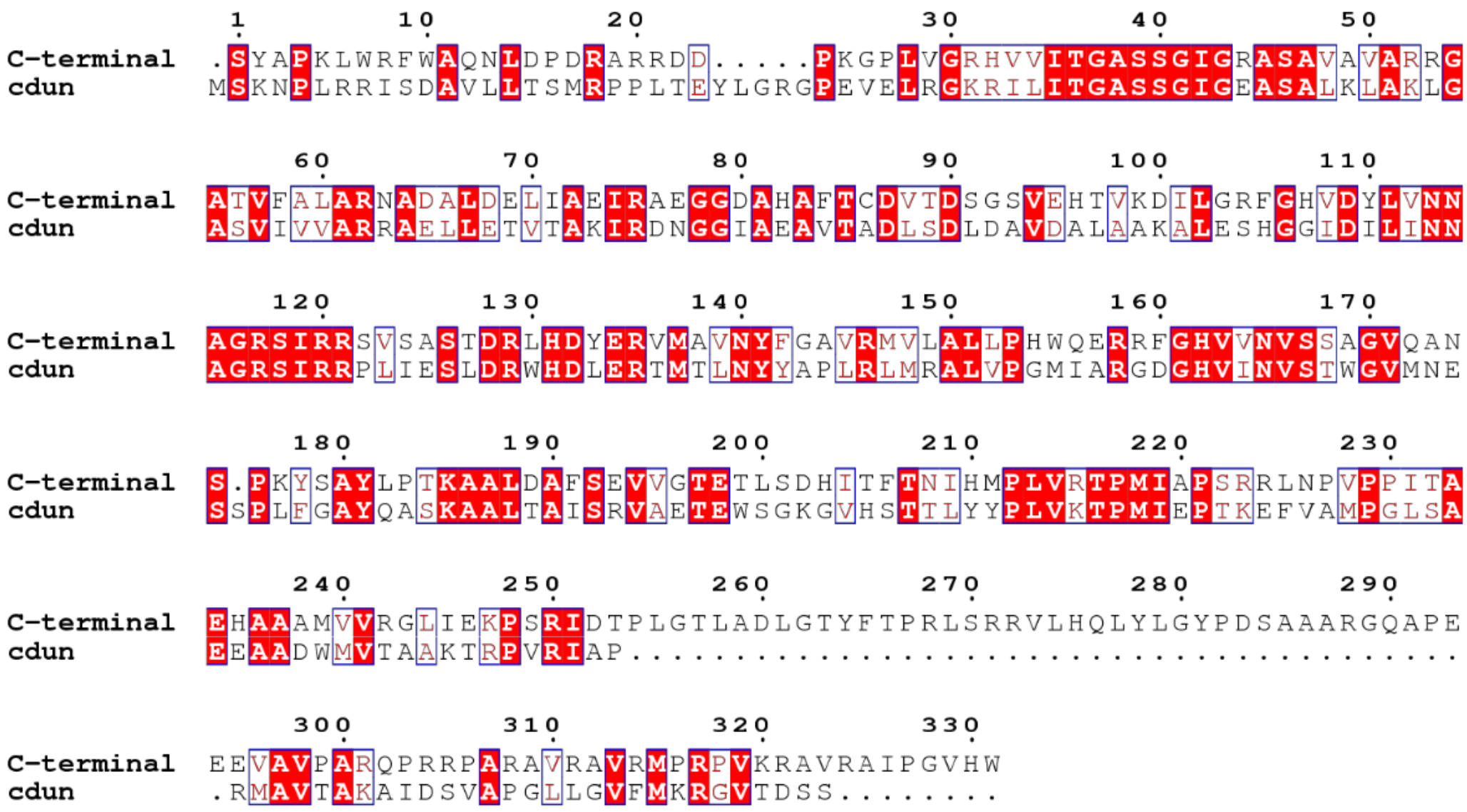

Figure S3 Amino acids sequence alignment between *Nb*Sal (*M. neoaurum*) and *Ps*Sal (*Pseudomonas sp.*).

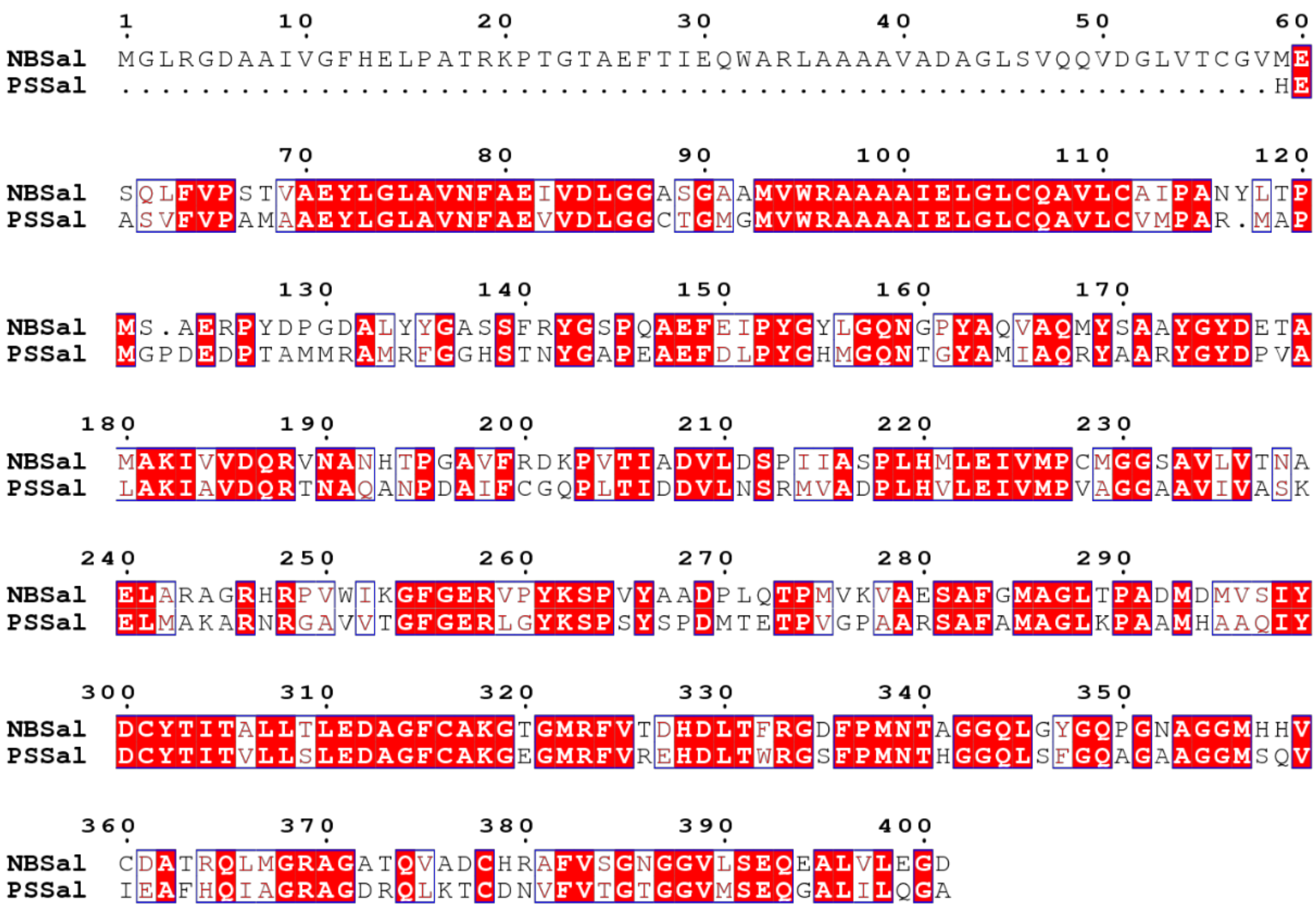

Figure S4 Optimization of biotransformation conditions for the improvement of ADD space-time yield by the engineered *M. neoaurum* NRRL B-3805$_{\Delta kshA1\text{-}opccR\text{-}sal\text{-}pL2\text{-}kstD1}$ under 30 g/L phytosterols. (A) Effect of different wet cell sediment. (B) Effects of different liquid volume. (C) The yields of ADD and AD under the conditions of 80g/L wet cell sediment and a liquid volume of 30 mL

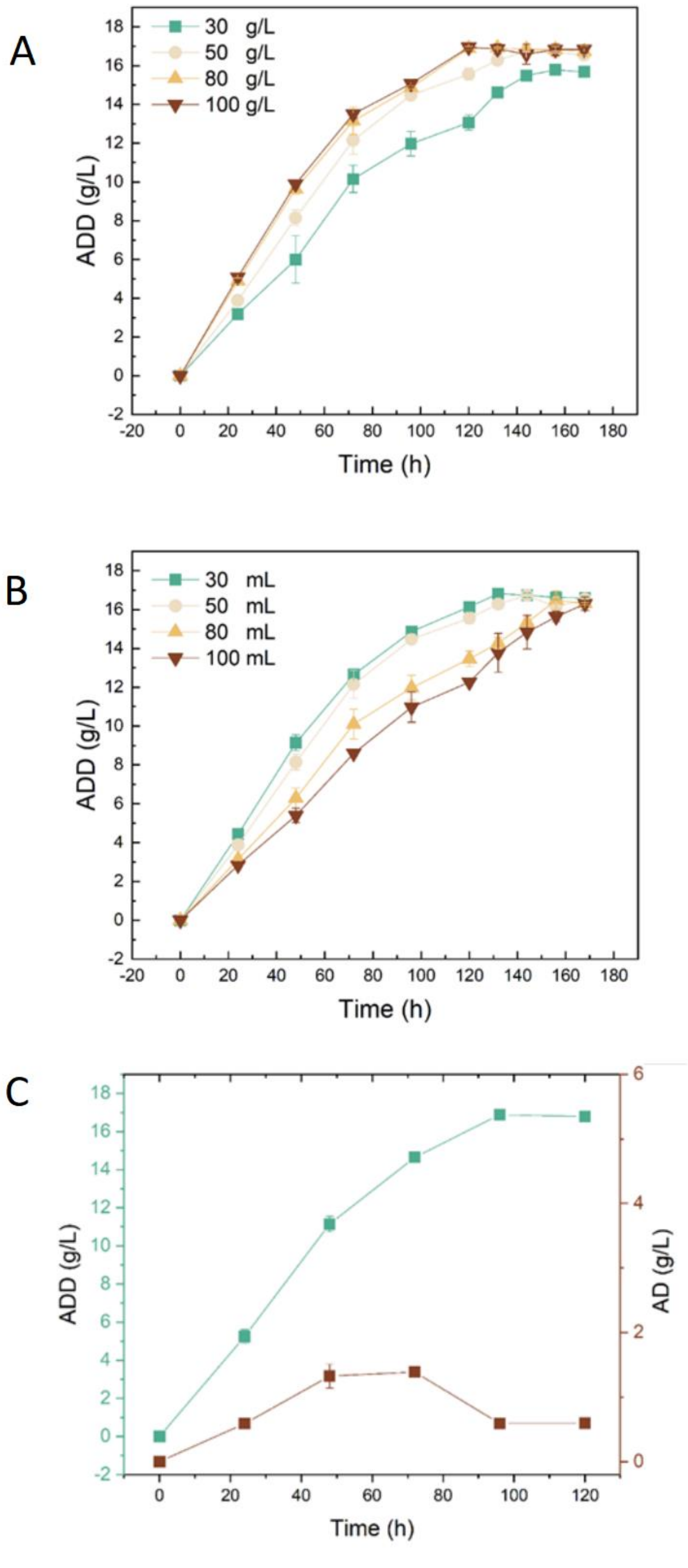

Supplement: Supplementary file 1 [file 253_2023_12847_MOESM1_ESM.pdf]
